# Gait Classification in Unilateral Cerebral Palsy

**DOI:** 10.3390/jcm8101652

**Published:** 2019-10-11

**Authors:** Stefanos Tsitlakidis, Axel Horsch, Felix Schaefer, Fabian Westhauser, Marco Goetze, Sebastien Hagmann, Matthias C. M. Klotz

**Affiliations:** 1 Clinic of Orthopedics and Trauma Surgery, Heidelberg University Hospital, Schlierbacher Landstrasse 200a, 69118 Heidelberg, Germany; axel.horsch@med.uni-heidelberg.de (A.H.); felix.schaefer@stud.uni-heidelberg.de (F.S.); fabian.westhauser@med.uni-heidelberg.de (F.W.); marco.goetze@med.uni-heidelberg.de (M.G.); sebastien.hagmann@med.uni-heidelberg.de (S.H.); 2 Clinic for Orthopedic & Trauma Surgery, Kepler University Hospital, Krankenhausstr. 9, 4020 Linz, Austria; Matthias.Klotz@kepleruniklinikum.at

**Keywords:** unilateral cerebral palsy, gait patterns, classification systems, lower extremity

## Abstract

As unilateral cerebral palsy represents a complex disorder, gait classification is difficult. Knowledge of the most frequent gait patterns and functional impairment is crucial for proper decision-making. This study analyzes the prevalence of gait patterns as well as the relation of different gait patterns and the Gross Motor Function Classification System (GMFCS). Eighty-nine patients were classified retrospectively using the GMFCS, the classification of Winters, Gage, and Hicks (WGH), and Sutherland et al. The distribution of GMFCS levels among the different gait patterns was analyzed using Chi-squared test. The most common subtypes were GMFCS level I, WGH type I, and recurvatum knee. Seventeen percent (WGH) and 59% (Sutherland) of the patients did not match any criteria. Applying both classifications complementarily reduced the number of unclassified patients significantly. There was no significant difference concerning the distribution of GMFCS levels or age among the different gait patterns. A combined use of various classification systems is beneficial for proper decision-making. Unclassified patients seem to be a heterogeneous subgroup concerning functional impairment. There is a need of further characterization of the unclassifiable gait patterns and the caused functional impairment. Instrumented gait analysis remains the gold standard and should be broadly used for future studies and in clinical practice.

## 1. Introduction

Cerebral palsy (CP) is a complex, permanent, and progressive neurological disorder, which leads to a wide variety of phenotypes and degrees of severity [1,2,3,4,5]. Movement and gait disorders cause higher energy expenditures, impaired mobility, and reduced autonomy [6,7,8,9]. Over time, the degeneration of ligaments and cartilage and even immobility may result from spasticity and contractures in patients with CP [4,10].

Patients with unilateral CP show different gait patterns [11,12]. The type of the gait abnormality is influenced by the primary brain injury, secondary deformities or compensatory mechanisms [4,5,13]. Gait patterns may even depend on walking speed and on the extent of asymmetry [5,13,14]. Gait classification is very demanding but of high clinical relevance. In order to initiate an appropriate treatment and to avoid secondary problems like degeneration of cartilage and immobilization, gait disorders should be detected as soon and as precisely as possible. In the past, numerous clinical classification systems have been developed [15].

In this context, the Gross Motor Function Classification System (GMFCS) is a well-established scale concerning the functional impairment, though disregarding the underlying gait disorder [5,16]. Winters et al. introduced a classification defining four morphological types to rate gait in unilateral CP taking the whole lower limb in the sagittal plane into account, and is most widely accepted and commonly used in preoperative decision-making as well as for postoperative evaluation [17,18,19,20]. As it is the biggest joint of the human body, the knee joint plays a key role in the development of gait disorders. Sutherland et al. showed that primary movement disorders of the knee can most frequently be observed in the sagittal plane. According to the findings of this study, they introduced a classification into four gait patterns in patients with bilateral CP [21]. The most common gait disorders in bilateral CP are crouch and stiff knee gait [11,22], whereas recurvatum knee gait is less frequent and has a prevalence of <10% [23].

There are only a few reports characterizing gait patterns in patients with unilateral CP [11,16,24]. Thus, little is known about prevalence and manifestations of the consecutive gait pathologies in unilateral CP.

Knowledge of the most frequent gait patterns and the caused functional impairment is crucial for proper decision-making and treatment of the underlying pathologic gait patterns [11,12,20]. Especially regarding unilateral CP, little is known about the most frequent gait abnormalities and the functional impairment in this considerably heterogeneous population.

The purpose of this retrospective cohort study was to analyze the prevalence of gait patterns in patients with unilateral CP using common classification systems. A further intent was to evaluate the relation between the different gait patterns and the GMFCS.

## 2. Patients & Methods

This study was conducted as a retrospective cohort study after approval by the local ethics committee of the Medical Faculty of the Ruprecht-Karls-University of Heidelberg (S-400/2017).

### 2.1. Patients

In this retrospective study patients showing unilateral CP (GMFCS I–III) without having any kind of treatment related to the lower extremity in the patient history were included. The data was extracted from the motion laboratory data base. 3D-instrumented gait analysis (IGA) was performed from 2006 to 2017 using a 120-Hz 9-camera system (Vicon, Oxford Metrics, Oxford, UK) and two piezoelectric force plates (Kistler, Winterthur, Switzerland). Reflective markers were applied to bony landmarks according to the protocol of Kadaba et al. [25]. All participants were asked to walk barefoot at a self-selected speed along a seven-meter walkway. Finally, 89 patients (40 female (f), 49 male (m)) matched the inclusion criteria (unilateral CP, no previous surgery of the lower limbs, no Botulinum–Toxin–A injections within the last 6 months, GMFCS I–II) with a mean age of 15.3 ± 9.6 years (4–58 years) were included in this study. No limitations or exclusion criteria concerning the patient’s age were applied, thus allowing the evaluation of possible relations between age and the different gait patterns.

### 2.2. Clinical Classification

The functional impairment and abnormal gait pattern of each patient were classified according to:

- Gross Motor Function Classification System (GMFCS) [26]
  - level I: walking indoors/outdoors (including running and jumping), climbing stairs without support
  - level II: walking indoors/outdoors, climbing stairs with a railing
  - level III: walking indoors/outdoors with assistive mobility devices
  - level IV: walking ability severely limited even with assistive devices; using wheelchairs most of the time
  - level V: impaired in all areas of motor function
- the classification system of WGH [17]
  - type I: plantar flexion of the ankle in the swing phase with equinus deformity at initial contact and adequate dorsiflexion in the stance phase
  - type II: persistent plantar flexion of the ankle during the stance and swing phases
  - type III: plantar flexion of the ankle, progression of proximal involvement, and more limited flexion of the knee during the swing phase
  - type IV: plantar flexion of the ankle, restricted motion of the knee joint, and limited flexion–extension of the hip
- the classification system of Sutherland et al. [21]
  - crouch gait: minimum knee flexion in stance phase > 30°
  - jump knee gait: first peak knee flexion > 30° followed by minimum flexion in single support from 10° to 20°
  - stiff knee gait: peak knee flexion in swing phase limited to a maximum of 45° or late peak knee flexion in more than 30% of the swing phase
  - recurvatum knee gait: knee extension > 0° in stance phase

### 2.3. Data Analysis

Data was structured using Microsoft Excel 2013 (Microsoft, Redmond, WA, USA) and analyzed using SPSS Version 21.0 (IBM, Chicago, IL, USA).

For descriptive statistics, the mean and standard deviation were calculated. For comparative statistics, a Chi-squared test was performed to evaluate the amount of unclassified gait patterns between the different classification systems and to evaluate the distributions of GMFCS levels among the different gait patterns. The analysis of differences in age between the several subgroups included two-tailed student’s *t*-test (for GMFCS levels) and ANOVA test (for WGH and Sutherland subgroups). The level of significance was set at *p* < 0.05.

## 3. Results

**Figure 1:**
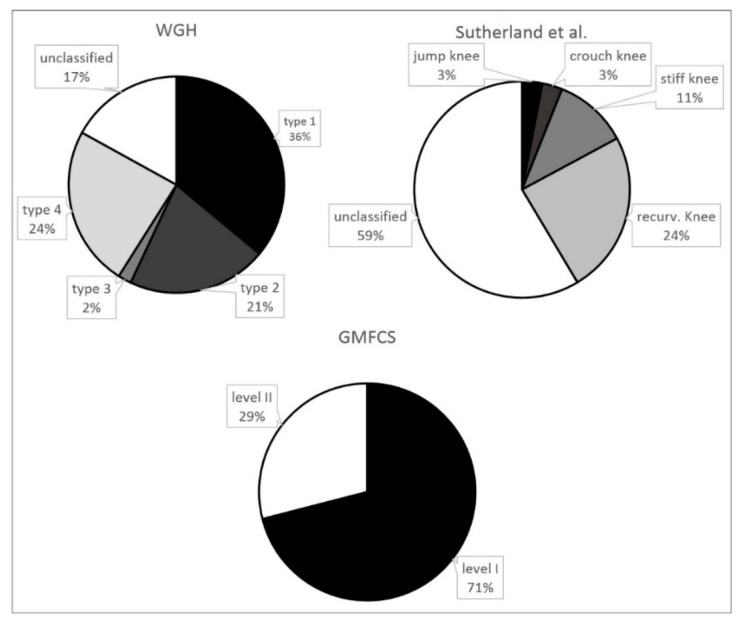
Prevalence of the several gait disorders according to the different classification systems (*n* = 89).

**Figure 2:**
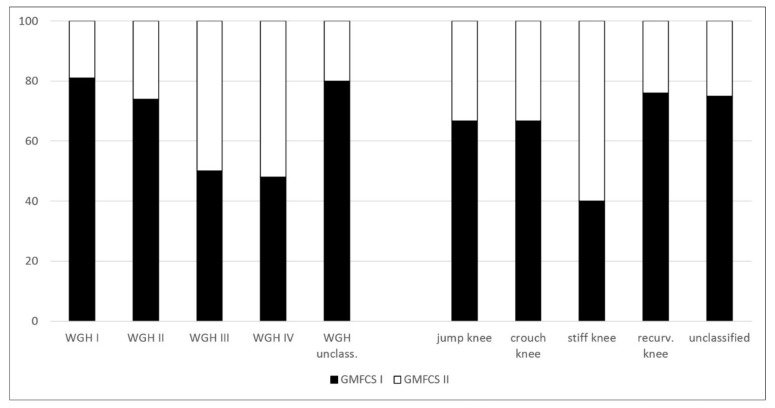
Percentage distribution of Gross Motor Function Classification System (GMFCS) levels within the different morphologic subgroups.

**Figure 3:**
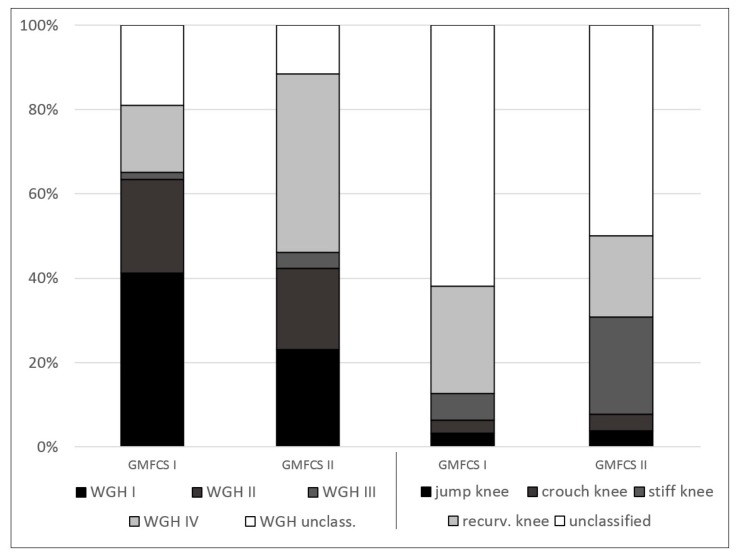
Percentage distribution of the different morphologic subgroups within Gross Motor Function Classification System (GMFCS) levels.

The percentage distribution of the 89 patients and the several subgroups of the different classification systems are provided in Figure 1. Figure 2 and Figure 3 show the distribution of GMFCS levels within the different morphologic subgroups and vice versa. Table 1 displays all patient distributions regarding the classification systems in association with each other as well as the mean age and sex distribution of all subgroups. The male to female ratio was 1.23:1.

71% of all patients showed only little functional impairment and the ability to walk and climb stairs without support (GMFCS I). According to the classification system of WGH, type I was predominant (36%), followed by type IV (24%). According to Sutherland et al., the most common gait pattern was recurvatum knee gait (24%), followed by stiff knee gait (11%).

Within the GMFCS levels, the most common morphologic subgroup among level I was WGH type I (41%), followed by WGH type II (22%) and WGH unclassified (19%) (Figure 3). Applying the classification system of Sutherland et al. on patients at GMFCS level I showed that most patients did not meet classification criteria (62%), followed by recurvatum knee gait (26%) (Figure 3). In GMFCS level II, the most common gait pattern according to WGH was type IV (42%), followed by type I (23%) and type II (19%) (Figure 3). Furthermore, according to Sutherland et al., GMFCS level II patients mainly showed unclassified gait (50%), followed by stiff knee gait (23%) (Figure 3).

Vice versa, with respect to the distribution of GMFCS levels within the WGH classification system, a gradual increase of GMFCS level II among patients from WGH type I to type IV is noticeable (19% among WGH type I vs. 52% among WGH type IV; Table 1 and Figure 2). Concerning the functional impairment, the unclassified patients showed the same distribution of GMFCS levels compared to WGH type I and II (Table 1). Concerning the distribution of GMFCS levels among the different gait patterns, according to Sutherland et al., the recurvatum knee gait showed the highest number of patients with GMFCS level I (76%, Table 1) whereas stiff knee gait was associated with the highest number of patients with GMFCS level II (60%, Table 1). The unclassified patients showed the same distribution of GMFCS levels compared to recurvatum knee gait (Table 1).

A considerable number of patients (17%) was not classifiable by the WGH classification system. Applying the classification system of Sutherland et al. as an optional and alternative classification, an even higher percentage (59%) of the patients did not meet the classification criteria. Interestingly, the majority of the unclassifiable patients showed little functional impairment (80% GMFCS I applying WGH; 75% GMFCS I applying Sutherland et al.; Table 1). Applying the WGH classification system in a second step on those patients that did not meet classification criteria according to Sutherland et al. revealed that the majority corresponded to WGH type I (48%) followed by WGH type IV (21%, Table 1). Vice versa, applying the classification system of Sutherland et al. on those patients that did not meet the criteria of the WGH classification system initially showed that 20% had recurvatum knee and 13% stiff knee gait.

Furthermore, the performed Chi-squared test revealed that there was no statistically significant differences between subtypes of the WGH classification system and between the subtypes of the classification system of Sutherland et al. concerning the distribution of GMFCS levels or vice versa between GMFCS levels concerning the distribution of WGH (*p* = 0.083) and Sutherland et al. (*p* = 0.213) subtypes. GMFCS level I and II showed the same percentage of unclassified patients.

Regarding possible differences with respect to age between the several subgroups, no statistically significant differences could be found (for GMFCS *p* = 0.082, for WGH *p* = 0.298; for Sutherland et al. *p* = 0.490).

Nine out of the 15 (WGH)/52 (Sutherland et al.) initially unclassified patients still did not meet any classification criteria (Table 1). These patients mainly had GMFCS level I (89%).

In total, by applying both classification systems, the amount of unclassifiable gait patterns could be reduced considerably by 41% (from 17% to 10% for WGH) and by 83% (from 59% to 10% for Sutherland et al.) (WGH vs. WGH/Suth. *p* = 0.187; Suth. vs. WGH/Suth. *p* < 0.05).

## 4. Discussion

Unilateral CP is a complex and heterogeneous disorder leading to a variety of pathologic gait patterns and functional impairment. Clinical classification of gait disorders in patients with unilateral CP remains challenging and still crucial for appropriate therapy planning.

The objective of this study was to analyze the prevalence of common abnormal gait patterns in patients with unilateral CP using different classification systems. A further intent was to assess the relation between these classifications and the functional impairment depicted by the GMFCS.

The most common subtypes in our cohort of patients with unilateral CP were GMFCS level I (63 out of 89), WGH type I (32 out of 89), and Sutherland unclassified or recurvatum knee gait respectively (52 and 21 out of 89) (Table 1). Our findings are similar to those of other authors [5,18,19]. A relation between those subtypes could be found. The most common subtypes amongst patients with a GMFCS level I were WGH type I and Sutherland unclassified and vice versa (Table 1).

The GMFCS is a well-known and established classification system with regard to functional impairment [16,26]. However, the main limitation regarding future treatment planning is the fact that the GMFCS does not depict the causative morphologic pathology and thus gives no information about treatment options. Our results suggest noticeable, though not statistically significant, differences regarding the distribution of morphological subtypes between GMFCS levels (Table 1, Figure 3). The number of unclassified patients were similar between GMFCS levels, whereas the 9 still unclassified patients mainly had GMFCS level I, indicating the complexity to classify patients with little functional impairment.

Concerning the relation between the morphological subtypes and function, the increasing number of patients with GMFCS level II from WGH type I to type IV represents the grading of involvement (Table 1, Figure 2). Our findings compare to those of Dobson et al. [5].

In a recent review by Papageorgiou et al., the WGH classification system was found to have excellent clinical applicability [15]. Nevertheless, there is a high number of patients with an unclassified gait pattern using the WGH classification system (Table 1). Several authors encountered the same issue (Riad et al. 23%; McDowell et al. 49%) and concluded in their kinematic-based study that the unclassified patients might not meet classification criteria, as they are less involved with respect to functional impairment and show non-detectable gait disorders [11,18,19]. As a result, Riad et al. renamed the unclassified patients and added a 0-group, as those patients showed the least deviation from the unimpaired gait in the IGA in order to modify and improve the validity of the WGH classification [18]. By contrast, our results suggest that the unclassified patients are a heterogeneous subgroup of different functional impairment. A considerable amount of the unclassified patients had GMFCS level II (20% WGH, 25% Sutherland et al.). The performed Chi-squared test indicated that there is no statistically significant difference between the subgroups of both classification systems concerning the distribution of GMFCS levels, suggesting that the unclassified patients are not less involved with regard to functional impairment compared to the classifiable patients.

The WGH classification system is widely used and was developed for patients with spastic hemiplegia, examining 46 patients mainly—but not exclusively—with CP [17,18,19]. However, due to the high number of unclassifiable patients, this classification system might not depict all gait disorders in patients with unilateral CP accurately. Furthermore, WGH types can be subdivided in further subtypes that would need different treatment regimen [20]. Those small differences were detected using IGA and analyzing EMG activation patterns [20,27].

Knowing that the classification of Sutherland et al. originally was developed for bilateral CP, this classification system was chosen as it includes further gait deviations of the knee joint [21]. The number of unclassifiable patients was 59%, even higher using the classification system of Sutherland et al. Apparently not all gait disorders can be depicted by the classification systems of WGH and Sutherland et al. leading to limitations in treatment planning.

By applying both classification systems complementarily, the number of unclassified patients could be reduced considerably, indicating that a single use of either classification system is insufficient. This might be due to the fact that both classification systems do not include every joint of the lower limb in every plane [12,17,18,21]. Furthermore, Wren et al. found intoeing and excessive stance-phase knee flexion in almost 50% of hemiplegic patients [11]. According to Papageorgiou et al., consensus among researcher groups was reached for six multiple joint patterns of patients with CP: “drop foot”, “true equinus”, “apparent equinus” “genu recurvatum”, “jump gait”, and “crouch gait” [15]. The WGH classification system mainly focuses on pathologies of the ankle joint and considers pathologies of knee and hip joint partially. Extending the number of possible gait patterns by using two morphological classification systems reduced the number of unclassified patients, thus improving the capability to classify gait disorders in patients with unilateral CP.

Accordingly, appropriate treatment planning is difficult, especially considering the natural progress of gait disorders. Gait impairment, e.g., caused by equinus, decreases with age, whereas an increase in rotational malalignment with age could be found [11,16]. However, our results revealed no statistically significant differences with respect to age between the different subgroups.

So far, it is questionable if specific treatment recommendations can be derived from clinical classification systems, as the often subtle gait disorders in patients with unilateral CP are difficult to be detected, quantified, and assessed [20,28,29]. Furthermore, none of the available clinical classification systems is able to depict all possible gait disorders and joint motion patterns accurately, still mainly focusing on sagittal plane kinematics [12,15]. In general, assigning individual patients to gait patterns rather than joint motion patterns may be a significant simplification of complex gait disorders leading to reduced validity, reliability, and applicability in clinical practice [15]. Furthermore, Gestel et al. and Dobson et al. stated that, as CP gait is often a mix of gait patterns, assigning patients into just one predominant or apparent pattern would jeopardize the usefulness of gait classification [5,30].

This circumstance emphasizes the need of the broad use of IGA investigating joint motion patterns. IGA to date still represents the crucial gold standard, as no other tool has achieved the accuracy and consistency including all joints and all dimensions of freedom [15,16,31,32]. Even though the broad implementation of IGA in clinical routine has been variable and controversial in the past, it is still essential for proper treatment decision-making. Algorithms for the automatic joint motion gait classification using available expert knowledge are options for refining the interpretability of IGA [30,33,34]. At least for those patients that do not meet any classification criteria or demonstrate more severe functional impairment, IGA applying joint motion patterns should be applied in a secondary step of pre-treatment diagnostics.

## 5. Conclusions

Currently available classifications systems are not sufficient to describe the gait patterns of patients with unilateral CP. A combined use of various clinical classifications (functional and morphological classification systems) is beneficial. There is a need for further characterization of the unclassifiable patients investigating separate joint motion patterns and the caused functional impairment in patients with unilateral CP to improve future clinical decision-making.

## Figures and Tables

**Table 1:** Prevalence of all gait disorders in association with each other (*n* = 89).

Classification System	*n*	GMFCS I	GMFCS II	WGH I	WGH II	WGH III	WGH IV	WGH Unclass	Mean Age (years)	Sex Distribution f/m
GMFCS										
I	63	-	-	/	/	/	/	/	13.9 ± 7.3	29:34
II	26	-	-	/	/	/	/	/	18.9 ± 12.9	11:15
WGH										
I	32	26	6	-	-	-	-	-	16.1 ± 9.6	17:15
II	19	14	5	-	-	-	-	-	11.4 ± 6.3	10:9
III	2	1	1	-	-	-	-	-	12.0 ± 0.0	1:1
IV	21	10	11	-	-	-	-	-	17.6 ± 12.3	7:14
unclassified	15	12	3	-	-	-	-	-	16.1 ± 7.6	5:10
Sutherland										
jump knee	3	2	1	1	0	0	1	1	10.7 ± 4.6	2:1
crouch knee	3	2	1	0	0	0	3	0	8.0 ± 5.3	1:2
stiff knee	10	4	6	2	1	1	4	2	17.8 ± 14.4	2:8
recurv. knee	21	16	5	4	12	0	2	3	16.5 ± 8.5	11:10
unclassified	52	39	13	25	6	1	11	9	15.1 ± 9.3	24:28

/, redundant value; n, number; f, female; m, male.
